# Highly Sensitive Detection of the Insecticide Azamethiphos by Tris(2,2′-bipyridine)ruthenium(II) Electrogenerated Chemiluminescence

**DOI:** 10.3390/s22072519

**Published:** 2022-03-25

**Authors:** Tesfaye Hailemariam Barkae, Abdallah M. Zeid, Guobao Xu

**Affiliations:** 1 State Key Laboratory of Electroanalytical Chemistry, Changchun Institute of Applied Chemistry, Chinese Academy of Sciences, 5625 Renmin Street, Changchun 130022, China; tesfaye@ciac.ac.cn (T.H.B.); dr_abdallah_zeid@mans.edu.eg (A.M.Z.); 2 School of Applied Chemistry and Engineering, University of Science and Technology of China, Hefei 230026, China; 3 Department of Chemistry, College of Natural & Computational Science, Wolkite University, Wolkite P.O. Box 07, Ethiopia; 4 Department of Pharmaceutical Analytical Chemistry, Faculty of Pharmacy, Mansoura University, Mansoura 35516, Egypt

**Keywords:** insecticide, azamethiphos, tris(2,2′-bipyridine)ruthenium(II), electrogenerated chemiluminescence, inhibitor

## Abstract

Azamethiphos (AZA) is an insecticide and neurotoxic agent that causes the inhibition of acetylcholinesterase (AChE). AChE is a vital enzyme for neurotransmission because it metabolizes acetylcholine neurotransmitter at the synaptic cleft and terminates synaptic transmission. It is worth mentioning that organophosphates and carbamates inhibit AChE. These AChE inhibitors bind to the active site of the enzyme and inactivate it, leading to paralysis and death. Herein, for the first time, we develop a sensitive, low-cost, and rapid electrogenerated chemiluminescence (ECL) system for the detection of AZA. The designed ECL sensor was applied for the highly sensitive detection of AZA with a wide dynamic range (from 0.1 μM to 1000 μM) and low detection limit of 0.07 μM (S/N = 3). The practical utility of the sensor demonstrates high recoveries (96–102%) in real samples of lake water and wastewater.

## 1. Introduction

Recently, aquaculture has become one of the most significant food industries, and its production has increased tremendously in the last three decades [1]. It is considered as the main source of primary production, having significant impact on the economic, social, and environmental aspects. Unfortunately, sea lice infestations account for the primary cause of economic loss [2]. It is estimated that 480 million USD is lost annually because of the decline in fish growth [3]. It is documented that azamethiphos (AZA) is used to control flies, sea lice infestations, mosquitoes, roaches, and other related public hygiene issues [4,5].

AZA is an insecticide and neurotoxic agent that acts by inhibiting acetylcholinesterase (AChE). AChE is found at neuromuscular junctions and in chemical synapses of the cholinergic type. Its activity serves to terminate synaptic transmission. However, AChE is targeted by inhibitors, such as organophosphates (OPs) and carbamates. These AChE inhibitors inactivate the enzyme by binding to the active site of AChE through phosphorylation and carbamylation. Consequently, the degradation of the neurotransmitter acetylcholine (ACh) occurred and resulted in the increment of Ach, producing excitation, paralysis, and death [6,7]. Thus far, the toxicological study and treatment of sea lice using AZA have been extensively studied [8,9,10,11,12,13]. To see the impact of AZA, different types of biomarkers have been used. In this regard, an AChE assay was extensively used to see and measure the impact of a sea lice treatment as the only biomarker and organophosphate toxicity [14]. Moreover, it has been reported that the cumulative effect of AZA leads to the accumulation of oxidized ferric in the tissue part of fish. This ultimately causes kidney damage, another piece of neurotoxicity evidence [15].

The detection of OP pesticide such as AZA was performed with traditional lab-based techniques, including chromatography [16,17,18], immunoassay [19,20,21], and enzyme inhibition [22,23]. For example, chromatography has limitations concerning its rapid and efficient detection because of the large-scale instrumentation, complexity in operation, the need for personal expertise, tedious sample preparations, longtime detection, and high cost. The enzyme inhibition approach is challenged by stability, activation, and reproducibility problems, and the immunoassay method is also limited by a high cost, expertise requirements, tedious assay procedures, and are time consuming. Interestingly, Babak Pashaei and his co-authors [24] designed dinuclear Ru(II) complexes with excellent performances for deep red light-emitting electrochemical cells (LECs) and electrogenerated chemiluminescence (ECL) OP sensors.

ECL is a type of luminescence in which light emission occurs owing to an electrochemical reaction. In line with this, the generated reactive intermediates undergo an electron transfer to generate an excited-state luminophore species, which, ultimately, emits light upon jumping to the ground state. ECL has the advantages of versatility, no external light sources, less background signal, a controllable potential, high sensitivity, and selectivity [25,26,27,28]. Importantly, the ECL of tris(2,2′-bipyridine)ruthenium(II) (Ru(bpy)_3_^2+^) has received much attention in immunoassays, clinical diagnoses, environmental analyses, and food monitoring, attributed to its good photochemical and electrochemical behaviors, excellent stability, and ECL emission in aqueous solutions. For instance, the Ru(bpy)_3_^2+^/tripropylamine (TPA) ECL has been extensively used for commercial immunoassays, where billions of dollars are obtained each year [28]. Both annihilation and the coreactant mechanism are followed to generate ECL signals. However, the coreactant mechanism has extensive applications because of the requirement of a single potential sweep, radical generated at a low potential, ECL generated in buffer solution, and being environmentally benign [27,29,30].

Herein, we develop Ru(bpy)_3_^2+^/AZA ECL system for the selective and sensitive detection of AZA. Impressively, this is the first report that describes an ECL method for AZA detection. Compared with other detection methods for OPs, such as chromatographic techniques, the Ru(bpy)_3_^2+^ ECL method has the advantage of a high sensitivity and simple instrument [31,32].

## 2. Experimental Section

### 2.1. Chemical and Material

Azamethiphos and Ru(bpy)_3_^2+^ were bought from Sigma-Aldrich (St. Louis, MO, USA). Metal salts were procured from Beijing Chemical Reagent Company (Beijing, China). To investigate the working phosphate-buffered solution of 0.1 M PBS, pH 8.5, series of pH were exploited by mixing stock solutions of NaH_2_PO_4_ and Na_2_HPO_4_. Stock solutions of AZA (10 mM) and (Ru(bpy)_3_)^2+^ (10 mM) were prepared. Chemicals and reagents consumed were all analytical grade and water used throughout the experiment was purified with Millipore system.

### 2.2. Apparatus

**Scheme 1:**
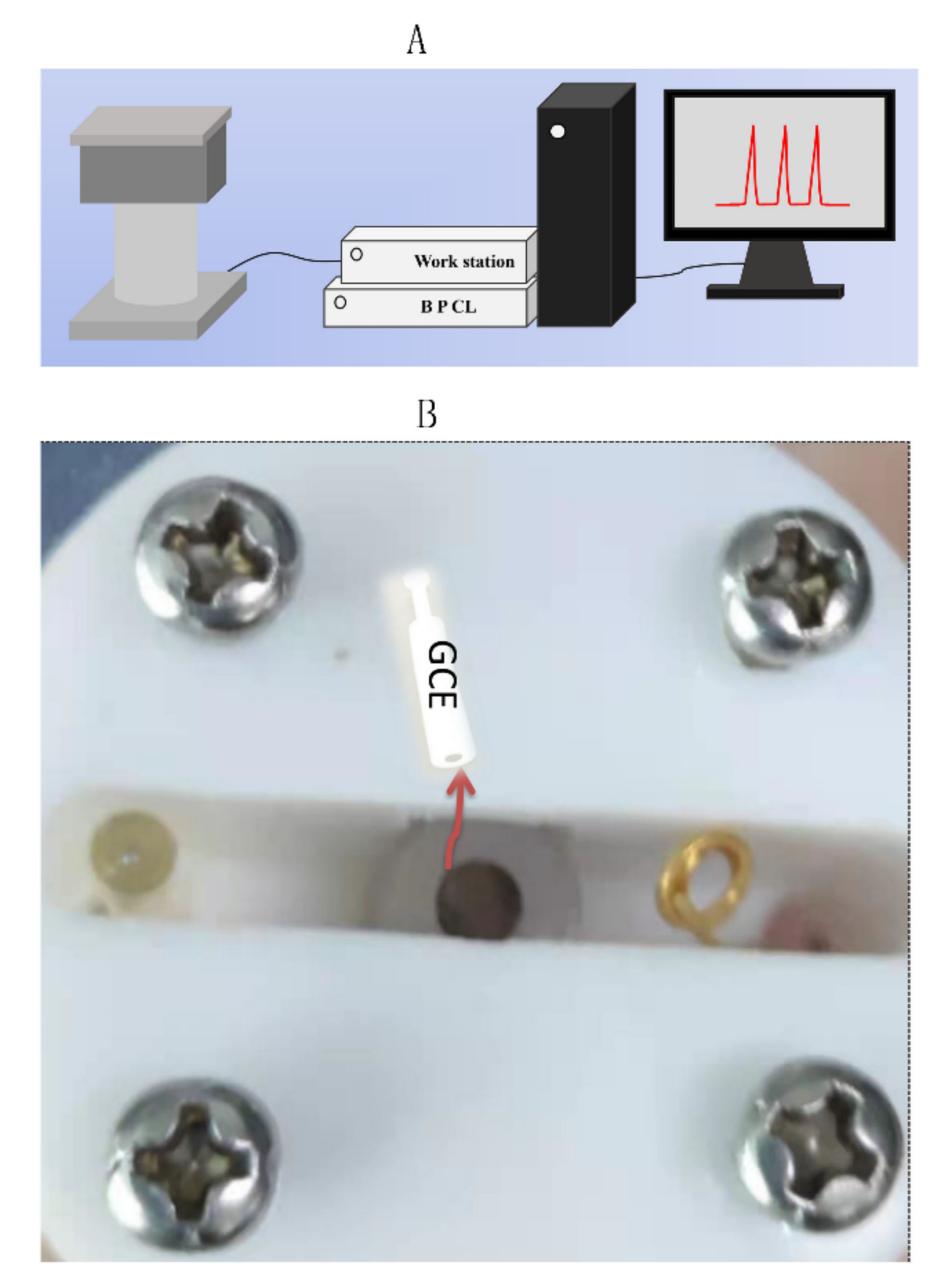
(**A**) Schematic diagram of ECL set up and (**B**) Front view of ECL cell.

The electrochemical workstation CHI 660C (provided by CH Instruments, Inc., Shanghai, China) was used to perform ECL experiment along with a homemade three-electrode cell with transparent bottom, where the photomultiplier tube (PMT) was adjusted to obtain the optimum ECL intensity (Scheme 1A). The typical usual setup consisted of glassy carbon electrode (GCE) as a working electrode, Pt wire as a counter electrode, and Ag/AgCl as a reference electrode (Scheme 1B). The diameter of glassy carbon was 3 mm. Aqueous slurries of 0.3 μm and 0.05 μm alumina were used each time before ECL measurement and then ultrasonicated and rinsed with ultrapure water. The potential was scanned from 0.0 to +1.5 V by setting the PMT at 900 V with a scan rate of 0.1 V/s to record ECL intensities. We measured the emission of all wavelengths except ECL spectrum experiments. To measure ECL spectra, wavelength filters ranging from 400 to 700 nm were placed between ECL cell and ECL detector.

### 2.3. ECL Detection

To detect AZA, different concentrations of AZA were mixed together with a specific volume (100 μL) of 10 mM Ru(bpy)_3_^2+^, and the pH was adjusted to pH 8.5 using 0.1 M PBS. The recorded ECL data were used to plot calibration graphs for detection purposes along with a potential scanned from 0.0 V to 1.5 V. The PMT was tuned at 900 V using a scan rate of 100 mV/s.

### 2.4. Real Sample Analysis Procedure

Waste water collected from the Changchun Institute of Applied Chemistry and Changchun south lake water were used as real samples. To detect AZA concentration, possible suspended impurities were first removed by filtration and the pH was adjusted with 0.1 M PBS (pH 8.5). Subsequently, 200 μL of the filtered and pH adjusted solution was mixed with 100 μL of 10 mM Ru(bpy)_3_^2+^ and 1700 μL 0.1 M PBS (pH 8.5). To perform recovery test, water sample was spiked with different concentrations of AZA and ECL detection was carried out based on the calibration curve.

## 3. Result and Discussion

### 3.1. Electrochemical and ECL Phenomena of Ru(bpy)_3_^2+^/AZA

**Figure 1:**
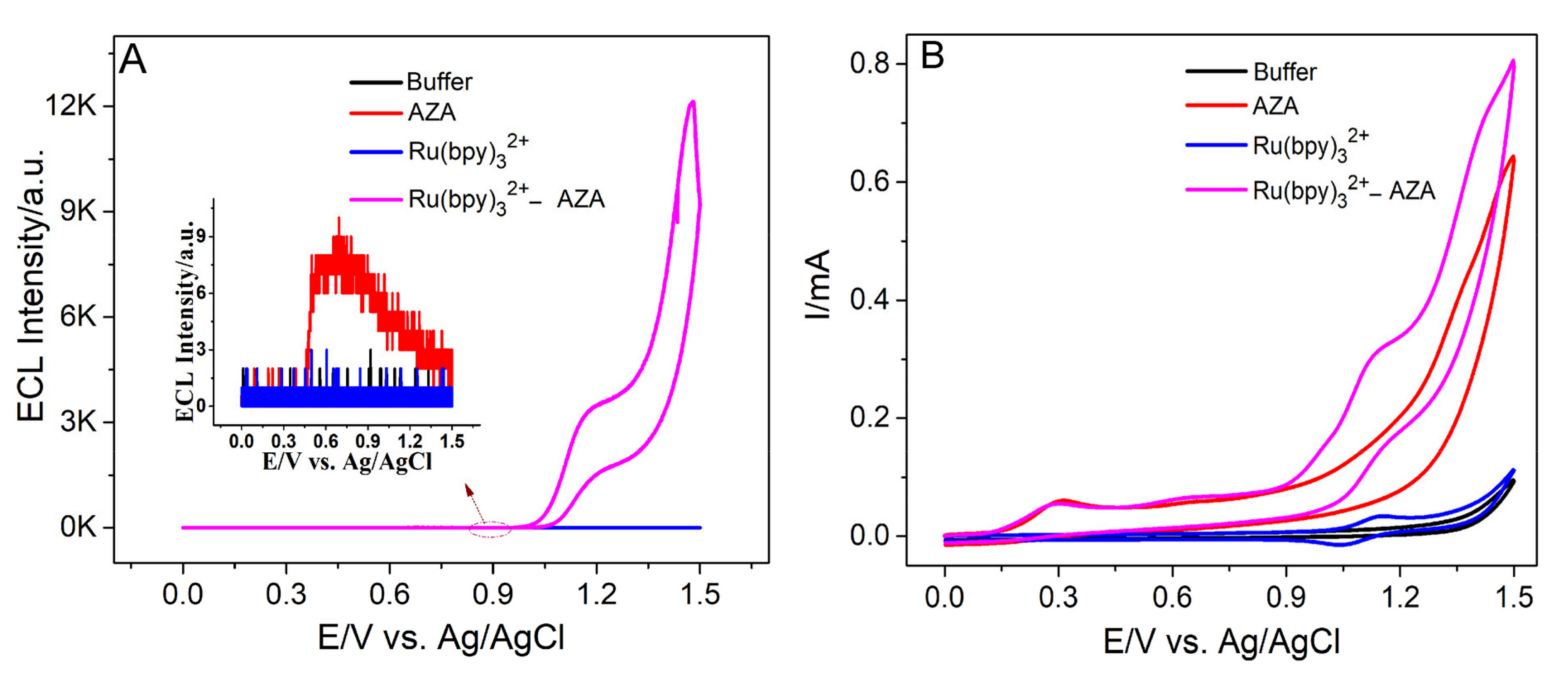
(**A**) ECL signal vs. potential and (**B**) cyclic voltammograms of buffer, 1 mM AZA, 0.5 mM Ru(bpy)_3_^2+^, 1 mM AZA/0.5 mM Ru(bpy)_3_^2+^ Carried out at GCE in 0.1 M PBS (pH 8.5); scan rate = 100 mV/s, PMT = 900 V. The inset is ECL curves of the buffer, AZA, and Ru(bpy)_3_^2+^.

To see the effect of the coreactant and reveal the electrochemical phenomenon, an investigation was conducted using ECL accompanied by cyclic voltammetry (CV). As illustrated in Figure 1A, the buffer (PBS), the coreactant AZA, and Ru(bpy)_3_^2+^ solutions hardly exhibited ECL emissions. Impressively, a remarkable ECL signal was observed after the addition of AZA to the Ru(bpy)_3_^2+^ solution. Thus, AZA was considered as an effective coreactant in improving the ECL signal of Ru(bpy)_3_^2+^.

Figure 1B illustrated the cyclic voltammograms of the buffer, AZA, Ru(bpy)_3_^2+^, and AZA/Ru(bpy)_3_^2+^ mixture. Notably, at a potential of approximately 0.32 V, AZA was oxidized and displayed irreversible oxidation peaks of AZA (red color), while Ru(bpy)_3_^2+^ demonstrated oxidation and reduction peaks at approximately 1.12 V and 1.04 V, respectively. Impressively, the addition of AZA to Ru(bpy)_3_^2+^ substantially increased the oxidation current and decreased the reduction current of Ru(bpy)_3_^2+^.

### 3.2. ECL Mechanism

**Figure 2:**
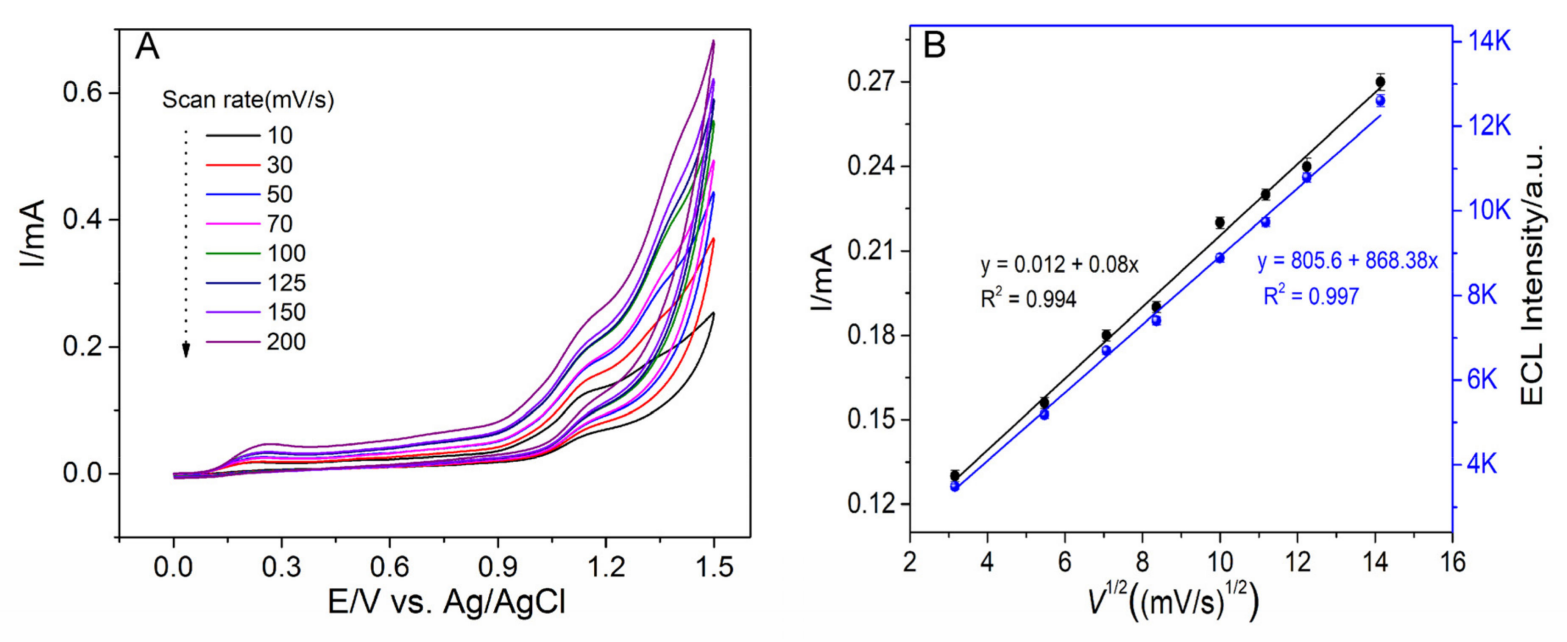
(**A**) Impact of scan rates vs. CVs and (**B**) Correlation of peak current and the ECL signal vs. square root of scan rate.

To propose the possible ECL mechanism, the influence of scan rates was investigated for the Ru(bpy)_3_^2+^/AZA system along with different scan rates of 10, 30, 50, 70, 100, 125, 150, and 200 mV/s, as illustrated in Figure 2. The square root of the scan rate correlated well with the peak current at 1.1 V and the ECL signal and, thus, the ECL reaction was controlled by diffusion [28,33,34,35,36].

**Figure 3:**
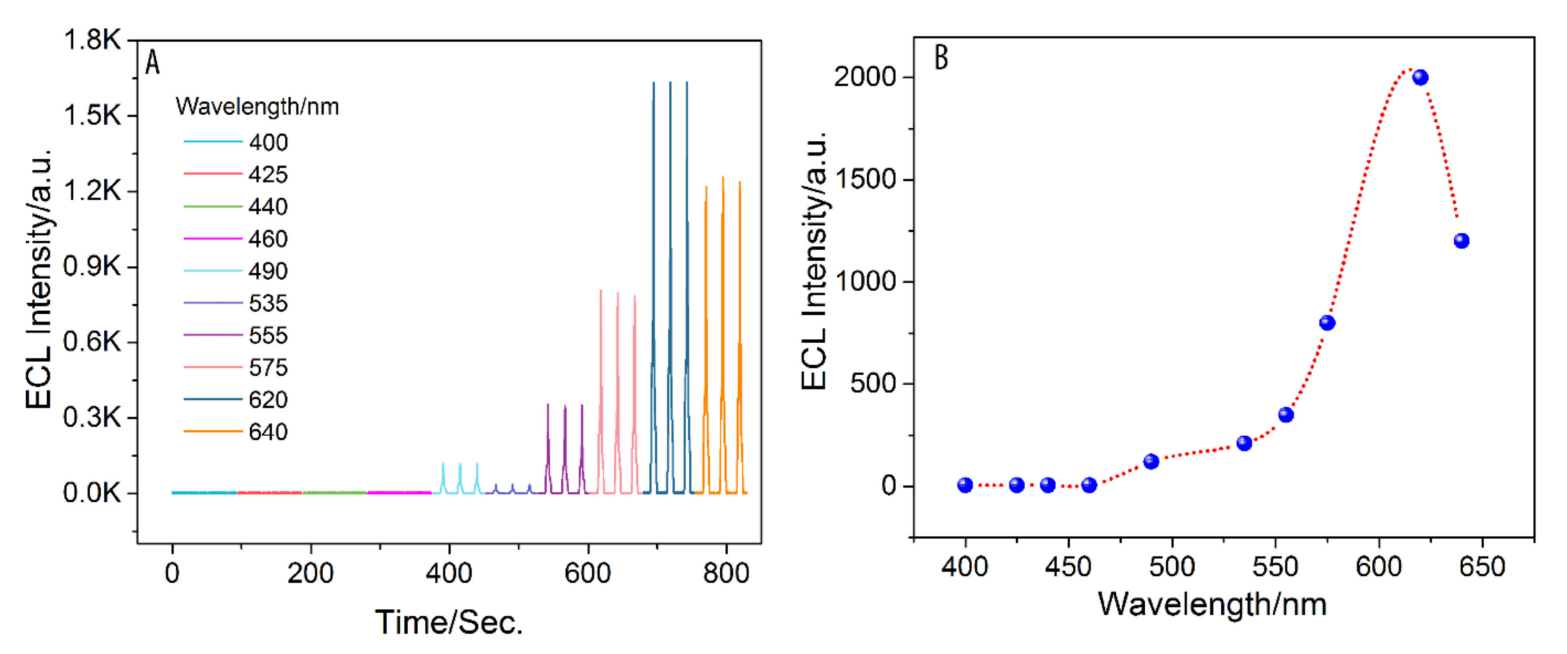
(**A**) Profile of ECL signal–time curve and (**B**) ECL emission spectrum of Ru(bpy)_3_^2+^/AZA sensor. The spectrum was recorded in the presence of 0.5 mM Ru(bpy)_3_^2+^ and 1 mM AZA in 0.1 M PBS (pH 8.5); PMT = 900 V.

To reveal the ECL mechanism, the ECL spectrum was measured by using wavelength filters of 400, 425, 440, 460, 490, 535, 555, 575, 620, and 640 nm. As shown in Figure 3, the wavelength of the maximum ECL emission was ~620 nm, which was consistent with that of Ru(bpy)_3_^2+^ ECL in the literatures [37,38,39]. The mechanism of the Ru(bpy)_3_^2+^/AZA ECL was proposed in Equations (1)–(5). Sweeping the potential anodically at GCE resulted in the oxidation of Ru(bpy)_3_^2+^ to Ru(bpy)_3_^3+^ (Equation (1)) and the oxidation of AZA to AZA^●^ (Equation (2)). AZA could also be oxidized by Ru(bpy)_3_^3+^ to AZA^●^ (Equation (3)). Thereafter, Ru(bpy)_3_^3+^ was reduced by AZA^●^ to produce Ru(bpy)_3_^2+^* (Equation (4)). Finally, Ru(bpy)_3_^2+^* emitted light after relaxation at λ_max_ of ~620 nm (Equation (5)).

Ru(bpy)_3_^2+^ − e → Ru(bpy)_3_^3+^ (1)

AZA − e → AZA^●^ (2)

Ru(bpy)_3_^3+^ + AZA → Ru(bpy)_3_^2+^ + AZA^●^ (3)

Ru(bpy)_3_^3+^ + AZA^●^ → Ru(bpy)_3_^2+^* + Products (4)

Ru(bpy)_3_^2+^* → Ru(bpy)_3_^2+^ + hν (5)

### 3.3. pH Optimization

**Figure 4:**
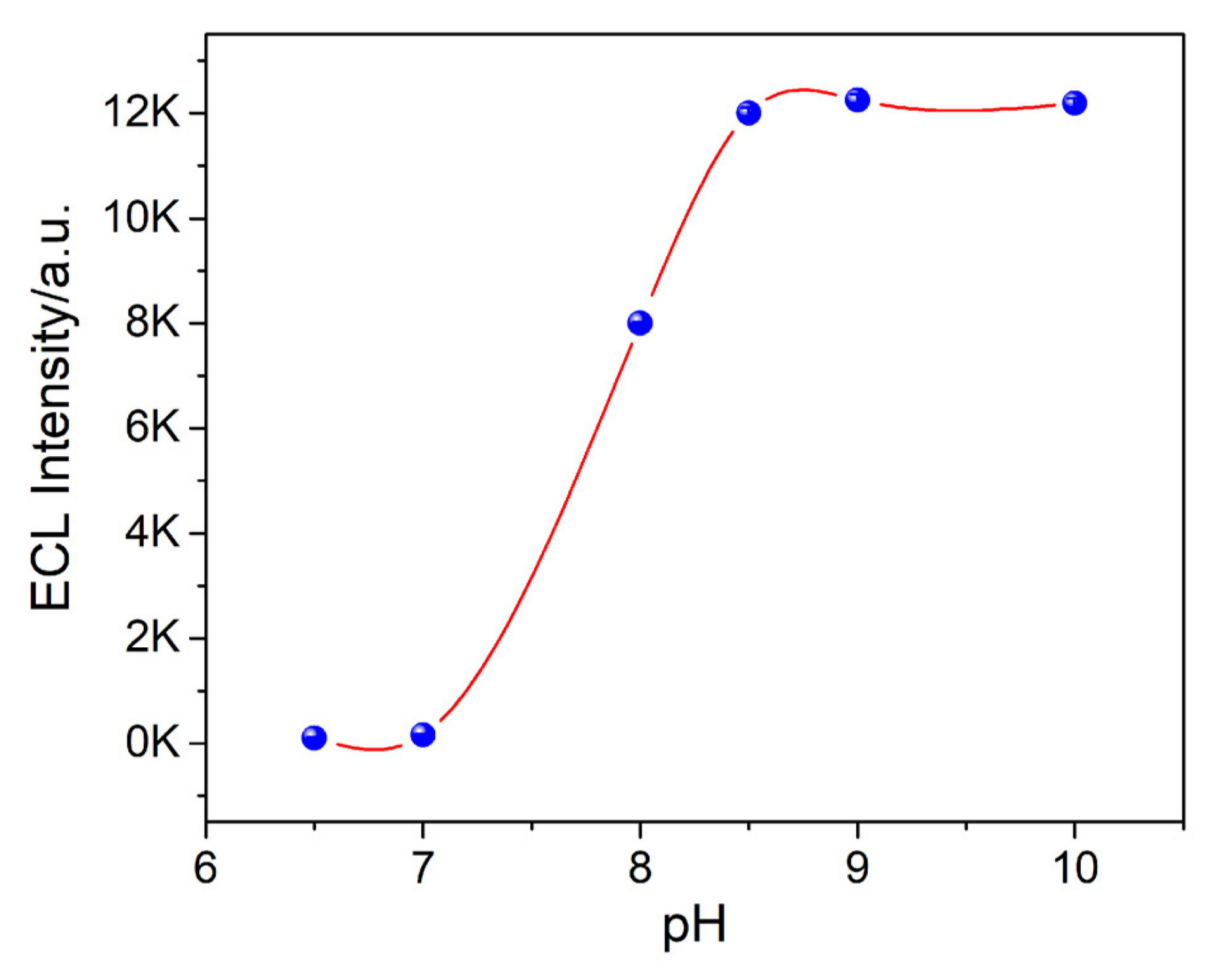
Effect of pH on ECL intensity of Ru(bpy)_3_^2+^/AZA system in 0.1 M PBS; 1.0 mM AZA and 0.5 mM Ru(bpy)_3_^2+^ were used in this study.

To reveal the impact of the pH on the ECL signal, a series of pHs ranging from 6.5 to 10 was investigated. As demonstrated in Figure 4, a very weak ECL intensity was observed at a pH ranging from 6.5 to 7.0. A significant increase in the ECL signal was observed when the pH increased from 8.0 to 10.0. Therefore, a pH of 8.5 was selected as the optimum pH for the next experiments.

### 3.4. Ru(bpy)_3_^2+^ Concentration Effect

**Figure 5:**
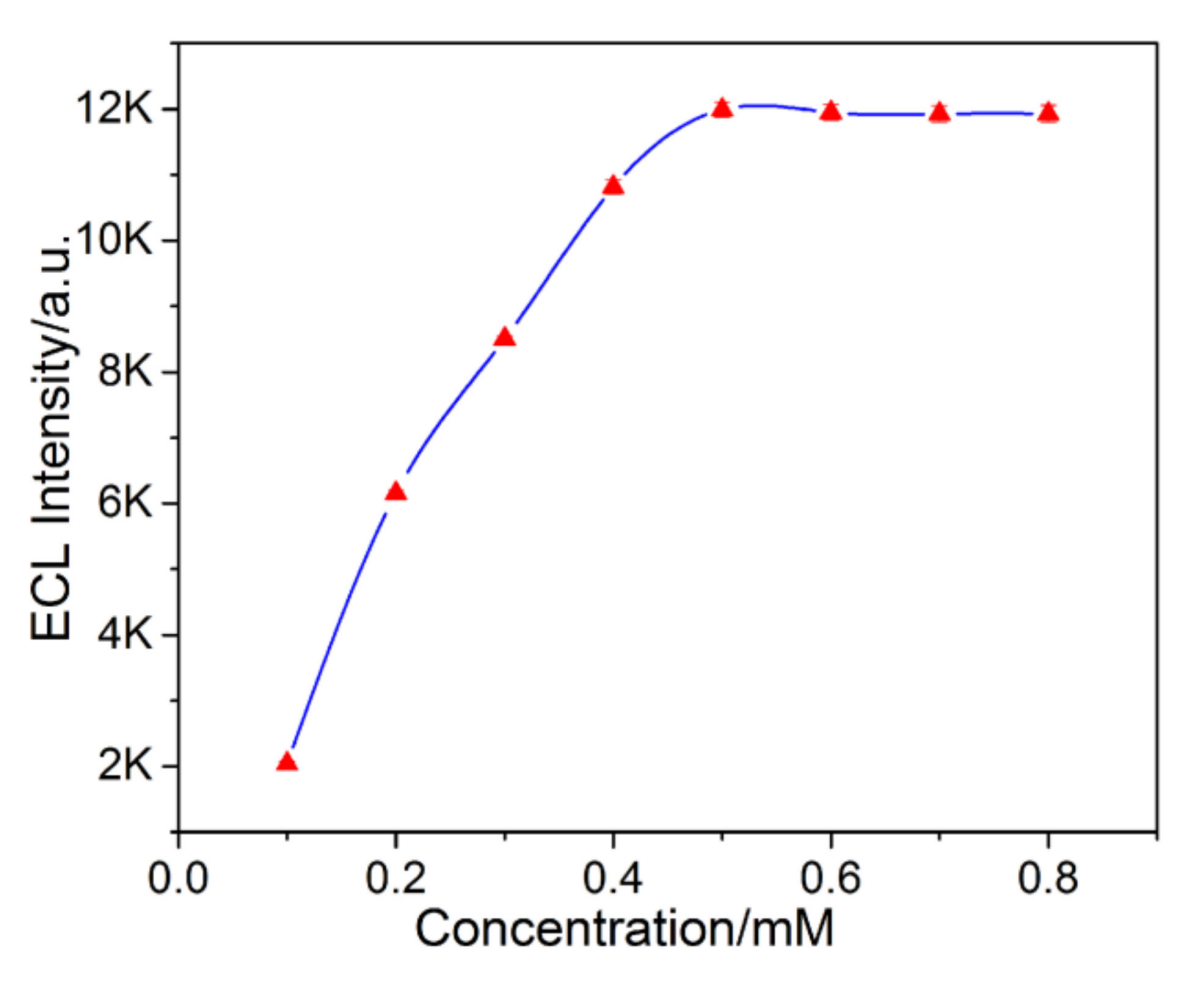
ECL intensity vs. the concentration of Ru(bpy)_3_^2+^. AZA (1.0 mM); PBS (0.1 M; pH 8.5); PMT = 900 V.

Various concentrations of Ru(bpy)_3_^2+^ (0.1–1.0 mM) were investigated to study the impact of Ru(bpy)_3_^2+^ concentration on the ECL signals of the Ru(bpy)_3_^2+^/AZA ECL platform. As clearly illustrated in Figure 5, a sharp increase in ECL intensities was observed proportionally with the increase in the Ru(bpy)_3_^2+^ concentration at a range of 0.1–0.5 mM. Almost no significant increment in the ECL signal was observed when the concentration of Ru(bpy)_3_^2+^ was higher than 0.5 mM. Thus, 0.5 mM of Ru(bpy)_3_^2+^ was used as the optimum concentration for subsequent experiments.

### 3.5. Detection of AZA

**Figure 6:**
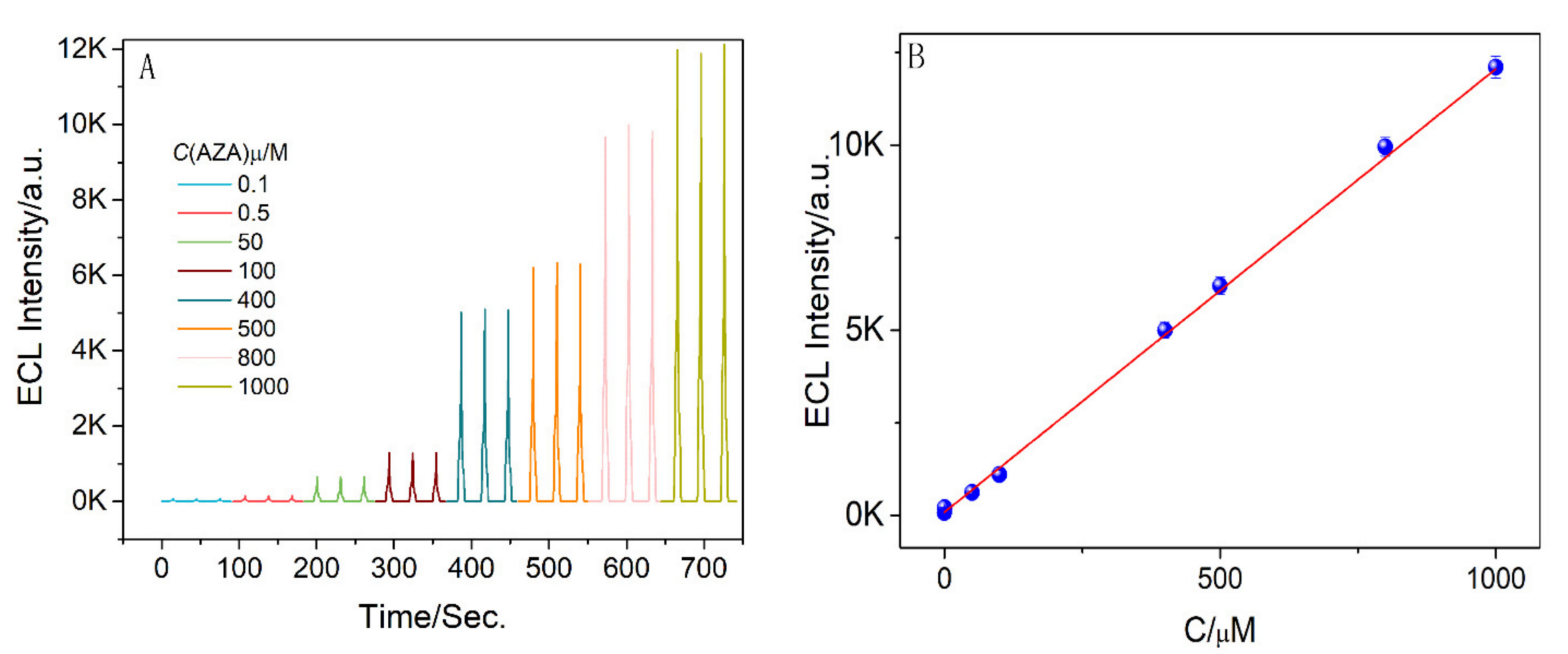
Detection AZA. (**A**) ECL–time profiles and (**B**) Calibration curve obtained for AZA detection from 0.1 to 1000 μM. Detection was employed in 0.1 M PBS (pH 8.5) in the presence of 0.5 mM Ru(bpy)_3_^2+^; PMT = 900 V.

**Figure 7:**
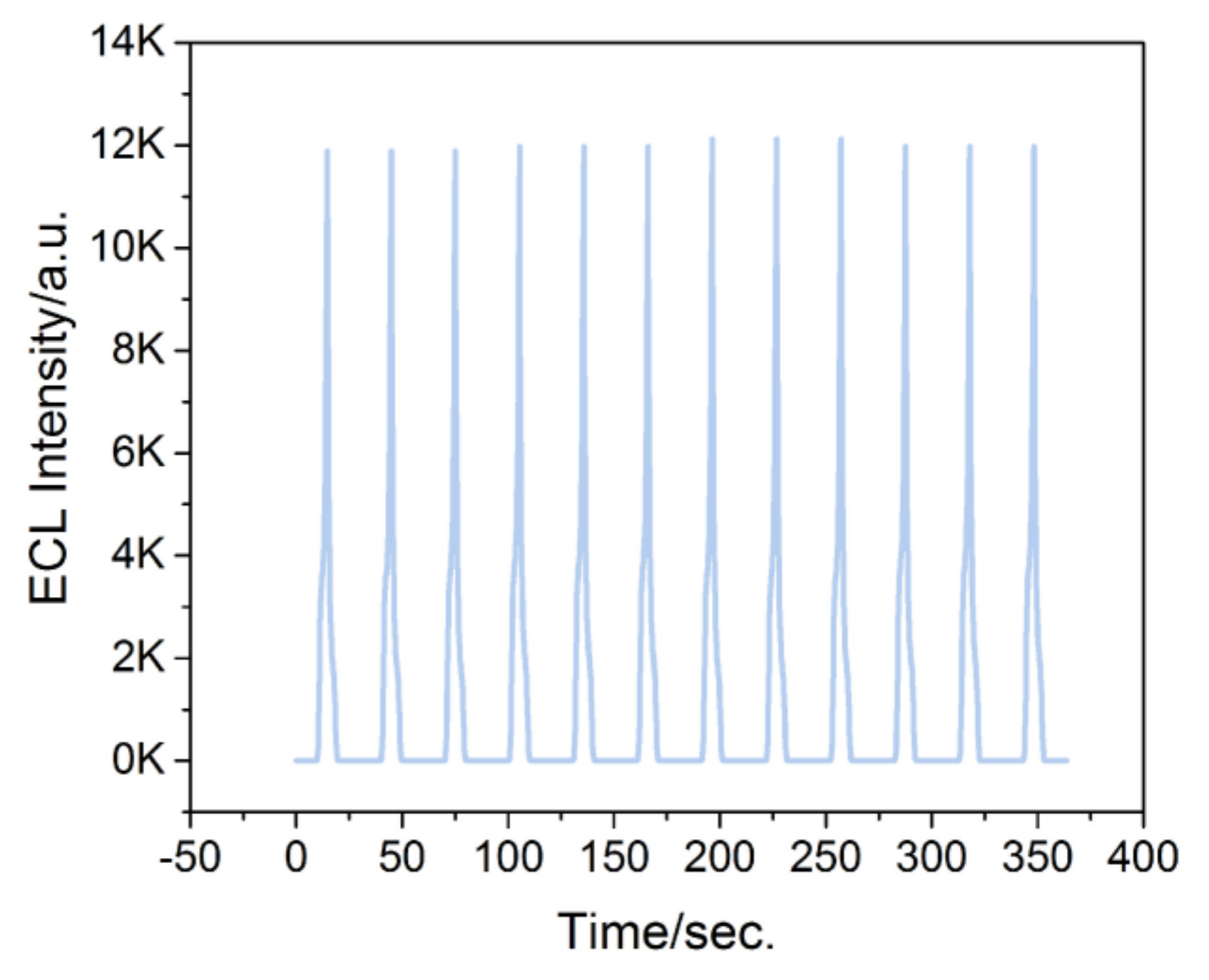
ECL–time curve profile. The detection was performed at pH 8.5 (0.1 M PBS) in the presence of 0.5 mM Ru(bpy)_3_^2+^ with 1.0 mM AZA; PMT = 900 V.

As illustrated from Figure 6A, as the concentrations of AZA increased, the ECL intensities increased proportionally. Thus, the developed ECL platform could detect and quantify AZA efficiently. Notably, a linear relationship between ECL intensities and the concentration of AZA (0.1–1000 μM) was obtained with the regression equation of *I_ECL_* = 11.97C (μM) + 81.39 (R^2^ = 0.996). The limit of detection (S/N = 3) reached 0.07 μM. The sensitivity of our method was comparable to that of the HPLC-UV method coupled with solid-phase microextraction, although our method did not use solid-phase microextraction [40]. Keeping the potential sweep anodically for twelve cycles and successive measurements of 1.0 mM AZA and 0.5 mM Ru(bpy)_3_^2+^, the obtained RSD was 3.31%, indicating the precision of the method (Figure 7).

### 3.6. Selectivity of the ECL Sensor Developed

**Figure 8:**
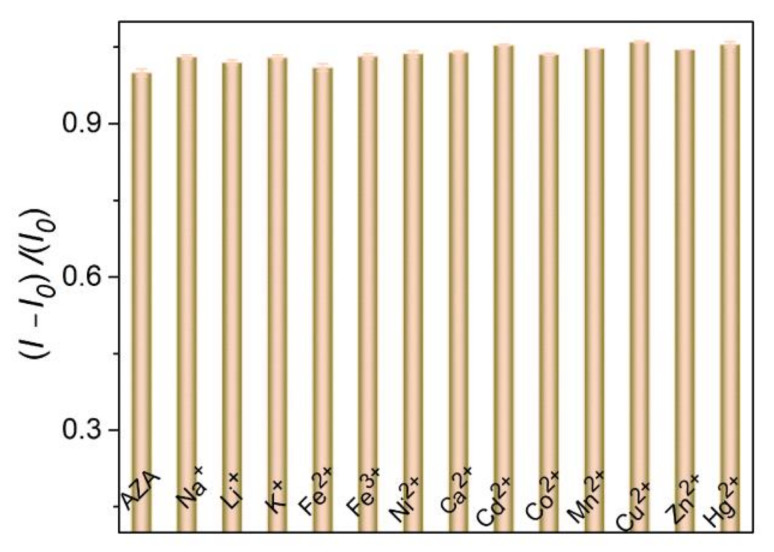
Selectivity toward other interfering species. *I*_0_ denotes the ECL emission of 0.5 mM Ru(bpy)_3_^2+^ with 2 μM AZA and *I* denotes the ECL signal after introduction of the interfering species; (*I* − *I*_0_)/*I*_0_ denotes the ECL change after introducing the interfering components. The concentration of the interfering species was 20 μM; 0.1 M PBS; pH 8.5.

Interfering species such as K^+^, Li^+^, Na^+^, Fe^2+^, Fe^3+^, Cu^2+^, Ca^2+^, Mn^2+^, Ni^2+^, Co^2+^, Zn^2+^, Cd^2+^, and Hg^2+^ were added to the solution, having 2 μM AZA and 0.5 mM Ru(bpy)_3_^2+^ to investigate the selectivity of the method. Notably, the difference of the ECL signal in samples along with all the species was not significant. These results further confirmed that the developed Ru(bpy)_3_^2+^/AZA ECL method had a good selectivity for the detection of AZA in the presence of other interferent species (Figure 8).

### 3.7. Analytical Application

The developed ECL platform was applied to detect AZA in water samples. We used both lake water samples from Changchun South Lake and waste water samples collected in our laboratory (Changchun Institute of Applied Chemistry). Since AZA is not found in water, the water samples were spiked with different concentrations of AZA. As illustrated in Table 1, the developed ECL method presented a high recovery. However, for samples that contained some related coreactants of Ru(bpy)_3_^2+^ ECL, the use of a separation technique (e.g., HPLC) or molecular recognition technique was needed to ensure a better selectivity.

## 4. Conclusions

AZA is an insecticide and neurotoxic OP agent, which acts by inhibiting AChE. A sensitive, cost-effective, and rapid ECL method was developed for the selective analysis of organothiophosphate insecticide, AZA. Remarkable ECL and electrochemical signals of the developed Ru(bpy)_3_^2+^/AZA sensor were obtained. We applied the developed ECL platform for the ultrasensitive detection of AZA in the range of 0.1 μM to 1000 μM, with a detection limit of 0.07 μM (S/N = 3). Impressively, the proposed ECL sensor was utilized practically to assay AZA in real water samples of lake water and wastewater and demonstrated high recoveries (96−102%). The study showed that ECL has promising potential for the detection of OPs.

## Figures and Tables

**Table 1:** Determination of AZA in Lake water and waste water samples.

Sample	Amount Added (μM)	Obtained (μM)	Recovery%	RSD
Lake water	0.5	0.49	98%	4.33
	10	10.2	102%	3.66
	50	48.5	97%	4.8
Waste water	0.5	0.48	96%	2.19
	10	9.9	99%	2.42
	50	50.5	101%	4.44

## Data Availability

All the data generated in the current research work were included in the manuscript.
